# Supplementary material for: Drivers and sites of diversity in the DNA adenine methylomes of 93 Mycobacterium tuberculosis complex clinical isolates
Source: eLife. 2020 Oct 27;9:e58542. doi: 10.7554/eLife.58542 (PMC7591249; doi:10.7554/eLife.58542)
Supplement: Transparent reporting form [file elife-58542-transrepform.docx]

***eLife’s* transparent reporting form**

We encourage authors to provide detailed information *within their submission* to facilitate the interpretation and replication of experiments. Authors can upload supporting documentation to indicate the use of appropriate reporting guidelines for health-related research (see [EQUATOR Network](http://www.equator-network.org/)), life science research (see the [BioSharing Information Resource](https://biosharing.org/)), or the [ARRIVE guidelines](http://www.plosbiology.org/article/info:doi/10.1371/journal.pbio.1000412) for reporting work involving animal research. Where applicable, authors should refer to any relevant reporting standards documents in this form.

If you have any questions, please consult our Journal Policies and/or contact us: [editorial@elifesciences.org](mailto:editorial@elifesciences.org).

**Sample-size estimation**

- You should state whether an appropriate sample size was computed when the study was being designed
- You should state the statistical method of sample size computation and any required assumptions
- If no explicit power analysis was used, you should describe how you decided what sample (replicate) size (number) to use

Please outline where this information can be found within the submission (e.g., sections or figure legends), or explain why this information doesn’t apply to your submission:

This work describes the diversity and characteristics of DNA methylomes among 93 globally distributed clinical isolates of *Mycobacterium tuberculosis* and *Mycobacterium africanum*. Therefore, most analyses of the dataset did not implement a statistical method that required power analysis.

We included strains spanning all seven major lineages of the *Mycobacterium tuberculosis* complex, publicly available sequenced clinical isolates. Isolates were included if raw kinetic data was available, and if the sequencing data and subsequent assembly passed our quality control criteria for *de novo* assembly.

**Replicates**

- You should report how often each experiment was performed
- You should include a definition of biological versus technical replication
- The data obtained should be provided and sufficient information should be provided to indicate the number of independent biological and/or technical replicates
- If you encountered any outliers, you should describe how these were handled
- Criteria for exclusion/inclusion of data should be clearly stated
- High-throughput sequence data should be uploaded before submission, with a private link for reviewers provided (these are available from both GEO and ArrayExpress)

Please outline where this information can be found within the submission (e.g., sections or figure legends), or explain why this information doesn’t apply to your submission:

The difference in sequencing kinetics between replicates is described

in Figure 1— figure supplement 1.

Inclusion criteria for biological samples are described in Materials & Methods under the subheading “Isolate acquisition and inclusion criteria.” Outliers and inclusion criteria for specific analysis are described in Materials & Methods, Results, Figure and Figure Supplement Captions.

In Materials & Methods, this information is under the subheadings “Genome assembly”, “Characterizing the kinetic error profile across technical replicates”, “Identification of hypervariable MTase motif loci”, “Heterogeneous methylation analysis”, “Identification of promoters”, “Bayesian classification of base-specific methylation status”, “Conserved hypomethylation patterns”, “Transcription factor binding motif scanning”, and “RNA-seq analysis”.

In Results, this information is under the subheadings “Anomalous methylation patterns in orphan MTase motif sites”, “Hypomethyalted MTase motif sites are rare yet remarkably consistent across isolates”, “Sequence context of most hypomethylated sites are consistent with transcription factor occlusion”, “DNA adenine Methylation is widespread and distinctly patterned at promoters”, and “HsdM promoter methylation is associated with transcription levels of downstream genes”.

Deposition of sequencing is described under the “Data Availability” section.

**Statistical reporting**

- Statistical analysis methods should be described and justified
- Raw data should be presented in figures whenever informative to do so (typically when N per group is less than 10)
- For each experiment, you should identify the statistical tests used, exact values of N, definitions of center, methods of multiple test correction, and dispersion and precision measures (e.g., mean, median, SD, SEM, confidence intervals; and, for the major substantive results, a measure of effect size (e.g., Pearson's r, Cohen's d)
- Report exact p-values wherever possible alongside the summary statistics and 95% confidence intervals. These should be reported for all key questions and not only when the p-value is less than 0.05.

Please outline where this information can be found within the submission (e.g., sections or figure legends), or explain why this information doesn’t apply to your submission:

Information regarding Statistical Reporting can be found in the captions for all Figures (1-8) and Table 1 of the manuscript, and their respective supplements.

(For large datasets, or papers with a very large number of statistical tests, you may upload a single table file with tests, Ns, etc., with reference to sections in the manuscript.)

**Group allocation**

- Indicate how samples were allocated into experimental groups (in the case of clinical studies, please specify allocation to treatment method); if randomization was used, please also state if restricted randomization was applied
- Indicate if masking was used during group allocation, data collection and/or data analysis

Please outline where this information can be found within the submission (e.g., sections or figure legends), or explain why this information doesn’t apply to your submission:

This work describes the diversity of DNA methylomes among globally distributed clinical isolates of *Mycobacterium tuberculosis* and *Mycobacterium africanum*. Therefore, there are no experimental groups based on treatment.

However, isolates grouped by distinct DNA methyltransferase genotypes displayed sequencing kinetics that indicated similar genome-wide heterogeneous methylation patterns. This information can be found in Results, under the subheadings “Diverse mutations drive DNA methyltransferase activity profiles” and “A subset of DNA MTase alleles drive constitutive intercellular mosaic methylation in *M. tuberculosis.*”

**Additional data files (“source data”)**

- We encourage you to upload relevant additional data files, such as numerical data that are represented as a graph in a figure, or as a summary table
- Where provided, these should be in the most useful format, and they can be uploaded as “Source data” files linked to a main figure or table
- Include model definition files including the full list of parameters used
- Include code used for data analysis (e.g., R, MatLab)
- Avoid stating that data files are “available upon request”

Please indicate the figures or tables for which source data files have been provided:

The raw Sequencing data for all clinical strains analyzed in this study are deposited under BioProject accessions PRJNA555636 and PRJEB8783.

Analysis scripts used in this study are publicly available on gitlab at: <https://gitlab.com/LPCDRP/dna-methylation>

Source data is provided for Figures 2, 3, 4, 6, 7, and 8, and Table 1. Source data includes sequencing kinetics of methyltransferase target loci, context sequences of target loci, output from a motif scanning tool, and summary statistics of target loci overlapping gene promoters.
